# Inhibition of spinal astrocytic c-Jun N-terminal kinase (JNK) activation correlates with the analgesic effects of ketamine in neuropathic pain

**DOI:** 10.1186/1742-2094-8-6

**Published:** 2011-01-24

**Authors:** Xiao-Peng Mei, Hui Zhang, Wei Wang, Yan-Yan Wei, Ming-Zhu Zhai, Wen Wang, Li-Xian Xu, Yun-Qing Li

**Affiliations:** 1 Department of Anesthesiology, School of Stomatology, Fourth Military Medical University, Xi'an, 710032, PR China; 2 Department of Anatomy, Histology and Embryology, K. K. Leung Brain Research Centre, Fourth Military Medical University, Xi'an, 710032, PR China

## Abstract

**Background:**

We have previously reported that inhibition of astrocytic activation contributes to the analgesic effects of intrathecal ketamine on spinal nerve ligation (SNL)-induced neuropathic pain. However, the underlying mechanisms are still unclear. c-Jun N-terminal kinase (JNK), a member of mitogen-activated protein kinase (MAPK) family, has been reported to be critical for spinal astrocytic activation and neuropathic pain development after SNL. Ketamine can decrease lipopolysaccharide (LPS)-induced phosphorylated JNK (pJNK) expression and could thus exert its anti-inflammatory effect. We hypothesized that inhibition of astrocytic JNK activation might be involved in the suppressive effect of ketamine on SNL-induced spinal astrocytic activation.

**Methods:**

Immunofluorescence histochemical staining was used to detect SNL-induced spinal pJNK expression and localization. The effects of ketamine on SNL-induced mechanical allodynia were confirmed by behavioral testing. Immunofluorescence histochemistry and Western blot were used to quantify the SNL-induced spinal pJNK expression after ketamine administration.

**Results:**

The present study showed that SNL induced ipsilateral pJNK up-regulation in astrocytes but not microglia or neurons within the spinal dorsal horn. Intrathecal ketamine relieved SNL-induced mechanical allodynia without interfering with motor performance. Additionally, intrathecal administration of ketamine attenuated SNL-induced spinal astrocytic JNK activation in a dose-dependent manner, but not JNK protein expression.

**Conclusions:**

The present results suggest that inhibition of JNK activation may be involved in the suppressive effects of ketamine on SNL-induced spinal astrocyte activation. Therefore, inhibition of spinal JNK activation may be involved in the analgesic effects of ketamine on SNL-induced neuropathic pain.

## Background

Spinal glial activation is both required and sufficient for neuropathic pain after nerve injury [1-4]. Previous studies have also shown that spinal astrocytes and microglia are two key players in the induction and maintenance of neuropathic pain [5-7]. Specifically, astrocytes play a pivotal role in the maintenance of spinal nerve ligation (SNL)-induced neuropathic pain [8-10]. Accumulating evidence shows that mitogen-activated protein kinases (MAPKs), activated in spinal glia, play an important role in signaling cascades of inflammatory mediators during nerve injury-induced neuropathic pain [10-14]. There are three major MAPK family members: extracellular signal-regulated kinase (ERK), p38, and c-Jun N-terminal kinase (JNK) [11]. Ligation of the 5^th ^lumbar nerve results in activation of all three MAPKs in spinal cord and dorsal root ganglion (DRG), but each one has its own expression time window and different cellular localization [8,10,15]. Activated p38 is specifically localized within microglia but not neurons or the astrocytes in the spinal dorsal horn early after the lesion, which suggests that p38 activation in spinal microglia is likely to have a substantial role in the early phase of neuropathic pain [15,16]. Compared with p38, the JNK pathway is specific for spinal astrocyte activation in SNL-induced neuropathic pain [8,17]. SNL-induced transient activation of JNK in DRG neurons is involved in inducing neuropathic pain, whereas persistent activation of JNK in spinal astrocytes seems to be critical for maintaining SNL-induced neuropathic pain [8]. In particular, inhibition of JNK activation could alleviate neuropathic pain after SNL [8,17]. Therefore, inhibition of the JNK pathway during activation of spinal astrocytes could be the underlying mechanism of action for analgesics.

Intrathecal ketamine has been reported to have an evident analgesic effect on neuropathic pain induced by nerve injury or other causes in animal and clinical studies [18-20]. A number of studies have been carried out to elucidate the mechanisms underlying this analgesic effect, but without satisfying output. Previous study [21] showed that both intraperitoneal and intrathecal ketamine inhibit SNL-induced allodynia; however, intrathecal but not intraperitoneal ketamine suppresses SNL-induced astrocytic activation as reflected by down-regulated glial fibrillary acidic protein (GFAP) expression. These results suggest that intraperitoneal ketamine could alleviate SNL-induced neuropathic pain via classical "neuronal-based" mechanisms but, in addition, "astrocyte-related" mechanisms may also be important in underlying the anti-allodynic effect of intrathecal ketamine. Interestingly, ketamine can inhibit lipopolysaccharide (LPS)-induced astrocyte activation [22], and can also decrease LPS-induced JNK phosphorylation (pJNK) to inhibit pro-inflammatory gene expression in macrophages [23]. Therefore, we hypothesized that inhibiting activation of JNK pathway might be involved in the suppressive effect of ketamine on SNL-induced spinal astrocyte activation.

In the present study, the expression and localization of SNL-induced spinal pJNK was detected by immunofluorescence. Then, the effects of ketamine on SNL-induced mechanical allodynia were confirmed by behavioral testing. Finally, changes in SNL-induced pJNK expression after ketamine administration were quantified using immunofluorescence histochemistry and western blot.

## Methods

### Animal preparation

Male *Sprague-Dawley *rats (180-200 g) were housed in plastic cages, and maintained on a 12:12 h light/dark cycle under conditions of 22-25°C ambient temperature with food and water available. All efforts were made to minimize animal suffering and to reduce the number of animals used. All experimental procedures received prior approval from the Animal Use and Care Committee for Research and Education of the Fourth Military Medical University (Xi'an, China), and the ethical guidelines to investigate experimental pain in conscious animals [24].

### Intrathecal implantation

Intrathecal implantation was performed by inserting a polyethylene (PE) tube (Becton Dickinson and Company, USA) directly into the subarachnoid space of the lumbar enlargement to inject drugs. Briefly, a midline incision (3 cm) was made at the back of the rat from the level of the 3^th ^thoracic vertebrae to the lower back, under pentobarbital anesthesia (45 mg kg^-1^, *i.p.*). A pre-measured length of PE-10 tube (I.D. 0.28 mm and O.D. 0.61 mm) was passed caudally from the T8 to the L3 level of the spinal cord, and 2 cm of the free ending was left exposed in the upper thoracic region. Only animals judged to be neurologically normal and that showed complete paralysis of the tail and bilateral hind legs after administration of 2% lidocaine (10 μl) through the intrathecal catheter were used for the following experiments. Rats were allowed to recover for a 3-5 d period before further use.

### Spinal nerve ligation

To create the rat SNL model, under pentobarbital anesthesia (45 mg kg^-1^, *i.p.*), the left transverse process of the L6 vertebra was first removed to expose the L4 and L5 spinal nerves. The L5 spinal nerve was then carefully isolated and tightly ligated with 6-0 silk thread [25]. The surgical procedure for the sham group was identical to that of the SNL group, except that the spinal nerve was not ligated. The animals were followed for 1 w before intrathecal drug administrations were administered from post operative day (POD) 8 to POD 10.

### Intrathecal drug administrations

S(+)-ketamine hydrochloride (Sigma, St. Louis, MO, USA) was dissolved and diluted with preservative-free normal saline solution for administration. Normal saline (0.9%) was used as the negative control. Animals were divided into 4 groups for administration: a Sham-Saline group (n = 10, a volume of 10 μl normal saline was injected into Sham rats), an SNL-Saline group (n = 10, a volume of 10 μl normal saline was injected into SNL rats), an SNL-ketamine group (n = 30; 10 for each of the 3 subgroups; 10 μl of 30, 100 or 300 μg kg^-1 ^ketamine was injected into SNL rats, respectively); and a Sham-ketamine group (n = 30; 10 for each of the 3 subgroups; 10 μl of 30, 100 or 300 μg kg^-1 ^ketamine was injected into Sham rats, respectively). Drugs and normal saline were injected intrathecally over 30 s, followed by a 10 μl flush of normal saline. The dosages of ketamine used in the present study were chosen based on previous research [18,21] and pilot experiments.

### Nociceptive behavioral testing

Animals were habituated to the testing environment for 3 d before baseline testing, and then were placed under inverted plastic boxes (30 × 30 × 50 cm^3^) on an elevated mesh floor and allowed to habituate for 30 min before the threshold testing. Briefly, a logarithmic series of 8 calibrated Semmes-Weinstein monofilaments (von-Frey hairs; Stoelting, Kiel, WI, USA) were applied to the ipsilateral hindpaws to determine the stimulus intensity threshold stiffness required to elicit a paw withdrawal response. Log stiffness of the hairs is determined by log10 (milligrams × 10) [4]. The 8 filaments had the following log-stiffness values (value in grams is given in parentheses): 4.17 (1479 mg), 4.31 (2041 mg), 4.56 (3630 mg), 4.74 (5495 mg), 4.93 (8511 mg), 5.07 (11749 mg), 5.18 (15136 mg), and 5.46 (28840 mg). The range of monofilaments (1.479-28.840 gm) produced a logarithmically graded slope when interpolating a 50% response threshold of stimulus intensity (expressed as log10 (milligrams × 10)) [26]. The behavioral responses were used to calculate the 50% paw-withdrawal threshold (PWT, absolute threshold), by fitting a Gaussian integral psychometric function using a maximum-likelihood fitting method, as described in detail previously [4]. This fitting method allowed parametric statistical analysis. Assessments were made before surgery for baseline value. Then, behavioral tests were performed once a day until POD 10. Intrathecal drug administrations were carried out 30 min before behavioral test from POD 8 to POD 10. All the PWT tests were performed in a double-blind manner.

### Rotarod testing

In order to assess whether the drugs used in the present experiment could influence motor function, which might influence the behavioral results, we performed rotarod tests on drug-administered but operation- and behavioral observation-free rats. Rats with no previous exposure to the rotarod test were placed on the Ugo Basile 7650 Rotarod accelerator treadmill (Ugo Basile, Varese, Italy) set at the minimal speed for training sessions of 1-2 min at intervals of 30-60 min. After this learning period, the animals were placed on to the rotarod at a constant speed of 25 *RPM*. As the animal took a grip on the drum, the accelerator mode was selected on the treadmill, *i.e. *the rotation rate of the drum was increased linearly at 20 *RPM*. Thereafter, the time was measured from the start of the acceleration period until the rat fell off the drum. The cut-off time was 30 s. The test was done 30 min after intrathecal ketamine or saline once a day for 3 days. The time that the animal remained on the rotarod was recorded and expressed as a percentage of its own baseline value.

### Immunofluorescent histochemical staining

After deep anesthesia was induced using pentobarbital (60 mg kg^-1^, *i.p.*), rats were perfused through the ascending aorta with 100 ml 0.9% saline followed by 500 ml 0.1 M phosphate buffer (PB, pH 7.3) that contained 4% paraformaldehyde and 2% picric acid. After perfusion, the L5 spinal segment was removed and post-fixed in the same fixative for 2-4 h and then cryoprotected for 24 h at 4°C in 0.1 M PB that contained 30% sucrose. Transverse frozen spinal sections (30 μm in thickness) were cut with a cryostat (Leica CM1800; Heidelberg, Germany) and collected serially into three dishes. Each dish contained a complete set of serial sections.

The sections in the first dish were rinsed in 0.01 M phosphate-buffered saline (PBS, pH 7.3) three times (10 min each), blocked with 2% goat serum in 0.01 M PBS that contained 0.3% Triton X-100 for 1 h at room temperature (RT, 20-25°C), and then used for immunofluorescent histochemical staining. The sections were incubated overnight at 4°C with rabbit anti-phosphorylated JNK (pJNK) antibody (1:1000; Cell Signaling Technology, Beverly, MA, USA). The sections were washed three times in 0.01 M PBS (10 min each) and then incubated for 4 h at RT with the secondary antibody: Alexa 488 donkey anti-rabbit IgG (1:500; Invitrogen, Carlsbad, CA).

Other primary antibodies used in this study were monoclonal antibodies: mouse anti-neuronal-specific nuclear protein (NeuN) (1:3000; Chemicon, Temecula, CA), mouse anti-glial fibrillary acidic protein (GFAP) (1:5000; Chemicon, Temecula, CA), and mouse anti-cd11b clone ox42 (1:500; Abcam, Cambridge, UK). For double immunofluorescence, sections were incubated with a mixture of two primary antibodies followed by a mixture of the two respective secondary antibodies (Alexa 488 donkey anti-rabbit IgG and Alexa 594 donkey anti-mouse IgG, 1:500; Invitrogen, Carlsbad, CA). Confocal images were obtained using a confocal laser microscope (FV1000; Olympus, Tokyo, Japan) and digital images were captured with Fluoview 1000 (Olympus). The excitation peak wavelength was 495 nm for Alexa 488 or 590 nm for Alexa 594, and the emission peak wavelength was 519 nm for Alexa 488 or 617 nm for Alexa 594.

The specificity of the staining was tested on the sections in the second dish by omission of the primary specific antibodies. No immunoreactive products were found on these sections. Sections in the third dish were used for Nissl staining (data not shown).

### Western blot

Animals were sacrificed after inducing deep anesthesia (pentobarbital, 60 mg kg^-1^, *i.p.*) and the L5 dorsal horns were quickly removed. The spinal dorsal horn was then dissected using the "open book" method [10]. Briefly, the L5 spinal cord segment was dissected according to the termination of the L4 and L5 dorsal roots. Then, the spinal segment was cut into a left and right half from the midline. Finally, the left half was further split into the dorsal and ventral horns at the level of the central canal. The selected region was homogenized with a hand-held pestle in sodium dodecyl sulfate (SDS) sample buffer (10 ml mg^-1 ^tissue), which contained a cocktail of proteinase and phosphatase inhibitors. The electrophoresis samples were heated at 100°C for 5 min and loaded onto 10% SDS-polyacrylamide gels with standard Laemmli solutions (Bio-Rad Laboratories, CA, USA). The proteins were electroblotted onto a polyvinylidene difluoride membrane (PVDF, Immobilon-P, Millipore, Billerica, MA, USA). The membranes were placed in a blocking solution, which contained Tris-buffered saline with 0.02% Tween (TBS-T) and 5% non-fat dry milk, for 1 h, and incubated overnight under gentle agitation with primary antibody rabbit anti-pJNK (1:1000; Cell Signaling Technology, Beverly, MA), rabbit anti-JNK (1:1000; Cell Signaling Technology) and mouse anti-β-actin (1:1000; Sigma, St Louis, MO, USA) respectively. Bound primary antibodies were detected with a horseradish peroxidase (HRP)-conjugated anti-rabbit or anti-mouse secondary antibody (1:10000; Amersham Pharmacia Biotech Inc., Piscataway, NJ, USA). Between each step, the immunoblots were rinsed with TBS-T. All reactions were detected by the enhanced chemiluminescence (ECL) detection method (Amersham). The densities of protein blots were analyzed by using Labworks Software (Ultra-Violet Products, UK). The densities of pJNK, JNK and β-actin immunoreactive bands were quantified with background subtraction. Squares of identical sizes were drawn around each band to measure density, and background near that band was subtracted. Since β-actin levels didn't change significantly after inflammation and nerve injury [27], we used β-actin levels as loading controls, and pJNK or JNK levels were normalized against β-actin levels.

### Quantification and statistical analysis

All data were collected and analyzed by researchers blinded to the surgery and reagents used.

Data from immunofluorescence were calculated as detailed in our previous report [28]. For quantification of pJNK-immunopositive cell profiles in spinal cord, five nonadjacent sections (30 μm) from L5 segments were selected randomly from each animal. In each group, 6 rats were used for statistical analysis. Images (450 × 338 μm^2^) of the medial two-thirds of the superficial dorsal horn (laminas I-III) were captured under a 20 × objective [8]. All positively stained cells in the area were evaluated using a computer-assisted image analysis program (MetaMorph 6.1), which set low and high thresholds for immunofluorescent intensity determined by the signal. The same configuration was used to measure cell areas in all experimental groups. The measured areas were transferred to Excel automatically for the following statistic analysis. MetaMorph 6.1 was calibrated to provide standardization of area measurements. A standardized field area was sampled arbitrarily from regions within randomly selected dorsal horn sections [8]. Data from immunofluorescence were expressed as fold change against that of the Sham-Saline (or Sham) group. ANOVA followed by the least significant difference test was used for statistical analysis.

Data from western blots are expressed as mean ± SD. Differences in changes of values over time of each group were tested using one-way ANOVA, followed by the least significant difference test. Data from the von-Frey test are presented as mean ± SD and were analyzed as the interpolated 50% threshold (absolute threshold) in log base 10 of stimulus intensity (monofilament stiffness in milligrams × 10). Repeated measures ANOVA (with Bonferroni confidence interval adjustment) was used and conducted for analysis. Data from the rotarod test are presented as mean ± SD, and repeated measures ANOVA (with Bonferroni confidence interval adjustment) was also used. All statistical analyses were performed using SPSS^® ^version 16.0 software (SPSS Inc., Chicago, IL, USA). *P *< 0.05 was considered statistically significant.

## Results

### SNL induces significant ipsilateral up-regulation of astrocytic pJNK in spinal dorsal horn

**Figure 1:**
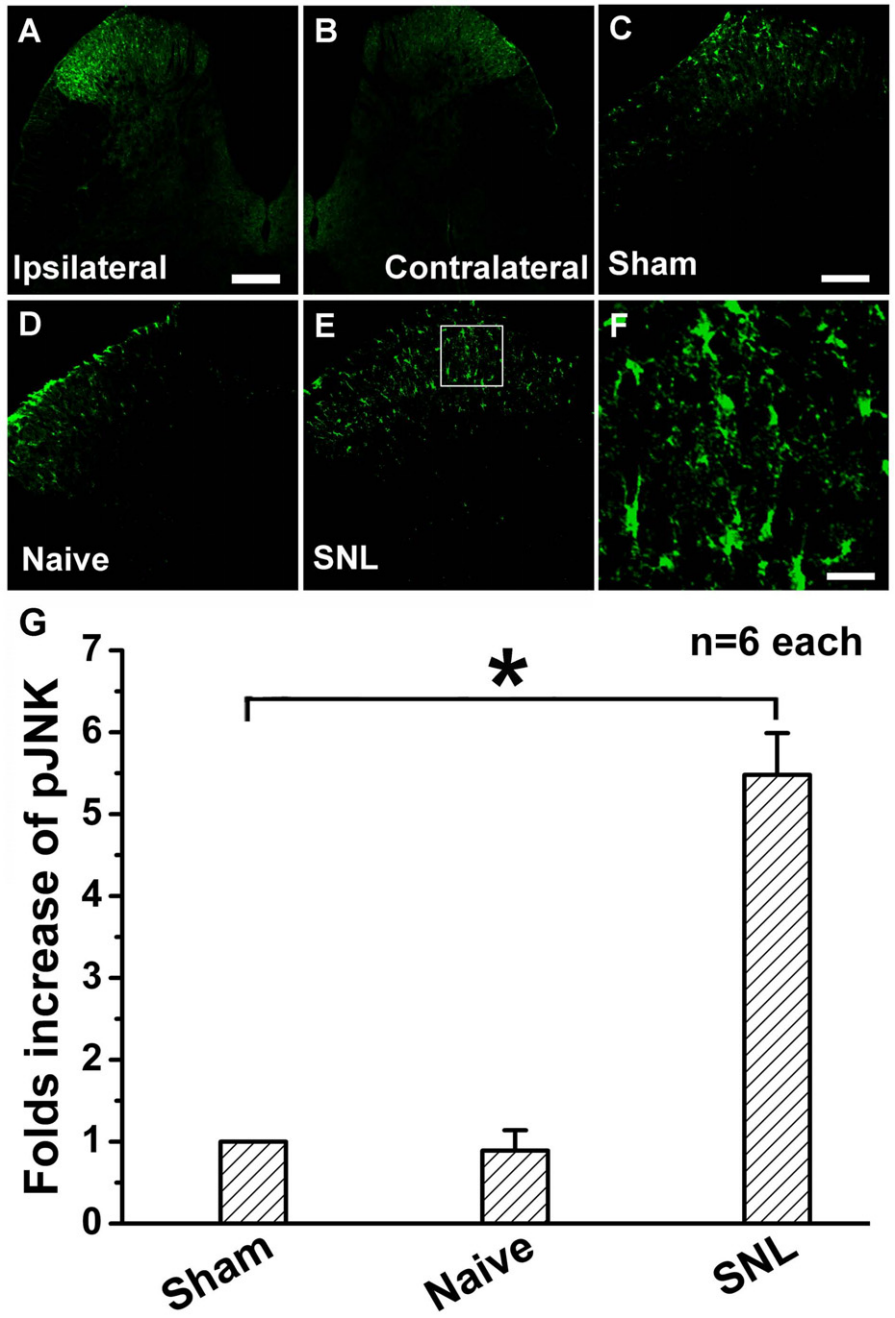
**Expression of pJNK in the rat spinal dorsal horn on postoperative day (POD) 10**. A, L5 spinal nerve ligation (SNL) induced a remarkable c-Jun N-terminal kinase phosphorylation (pJNK) up-regulation in the ipsilateral lumbar spinal dorsal horn. B, expression of pJNK in the contralateral lumbar spinal dorsal horn. C and D, expressions of pJNK in the ipsilateral lumbar spinal dorsal horn in Sham-Saline and naïve groups. E, up-regulation of pJNK in the ipsilateral lumbar spinal dorsal horn after SNL. F, a high-magnification image from the rectangle area of E shows a high density of pJNK-immunoreactive staining in the ipsilateral lumbar spinal dorsal horn after SNL. G, statistical analysis of pJNK expression in different groups. * indicates a statistically significant difference (*P *< 0.05) between groups. Six rats in each group. Scale bars: 200 μm in A and B, 100 μm in C, D and E, 20 μm in F.

SNL induced a marked pJNK up-regulation in ipsilateral spinal cord (Figure 1A), especially in the superficial dorsal horn, while few pJNK-immunopositive cells could be detected in contralateral spinal dorsal horn 10 days after SNL (Figure 1B). There was no significant difference in pJNK expression in spinal dorsal horn between Sham-Saline (Figure 1C) and naïve (Figure 1D) groups. However, compared with that of the Sham-Saline group, SNL induced a much higher expression of pJNK in the spinal dorsal horn (Figure 1E), indicated by increased density of immunoreactive staining in high-magnification images (Figure 1F).

**Figure 2:**
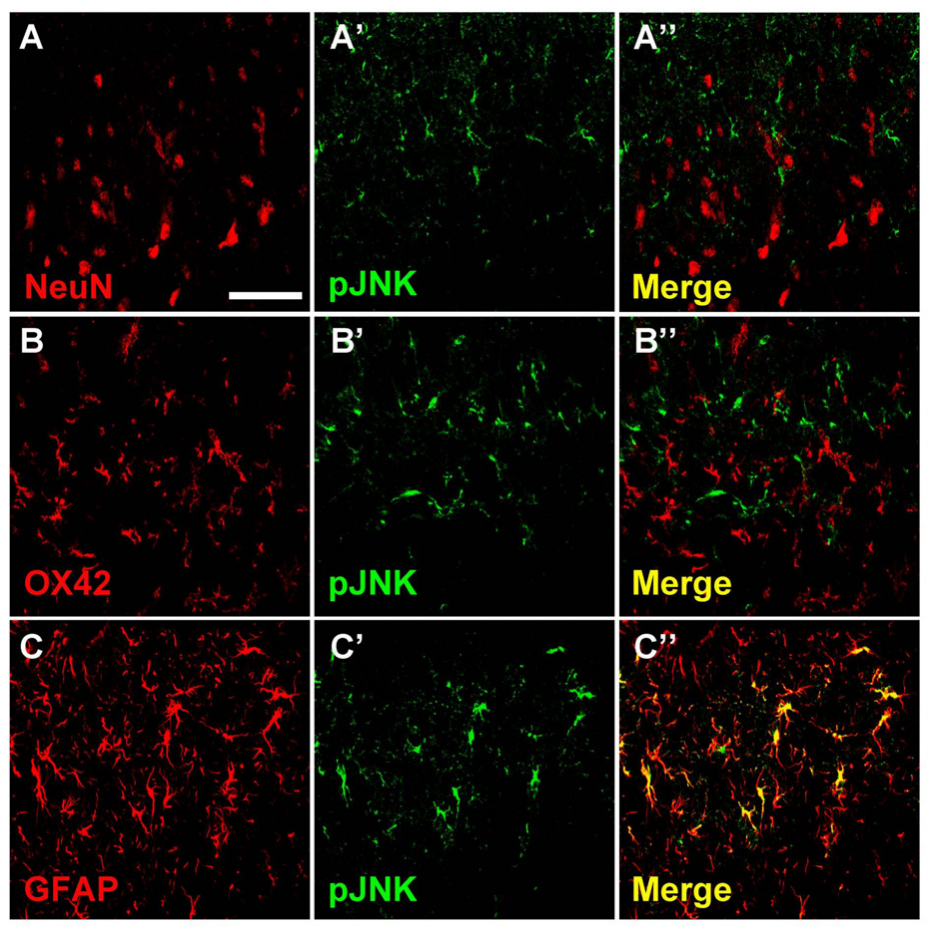
**Cellular localization of pJNK expression in spinal dorsal horn after SNL**. High-magnification images demonstrated the results of double immunofluorescent histochemical staining with NeuN and pJNK (A-A''), with OX-42 and pJNK (B-B''), or with glial fibrillary acidic protein (GFAP) and pJNK (C-C''). Scale bar: 50 μm.

In order to detect the cellular localization of pJNK expression, double immunofluorescent staining with antibodies against pJNK and the neuronal marker NeuN, the microglial specific marker OX42, or the astrocytic specific marker GFAP was performed, respectively. No colocalization could be observed either between pJNK and NeuN (Figure 2: A-A''), or between pJNK and OX42 (Figure 2: B-B''), which suggests that neither neurons nor microglia express pJNK in spinal dorsal horn 10 days after SNL. However, all pJNK-positive cells were GFAP-positive astrocytes (Figure 2: C-C'').

### Effects of ketamine on motor functions indicated by rotarod testing

**Figure 3:**
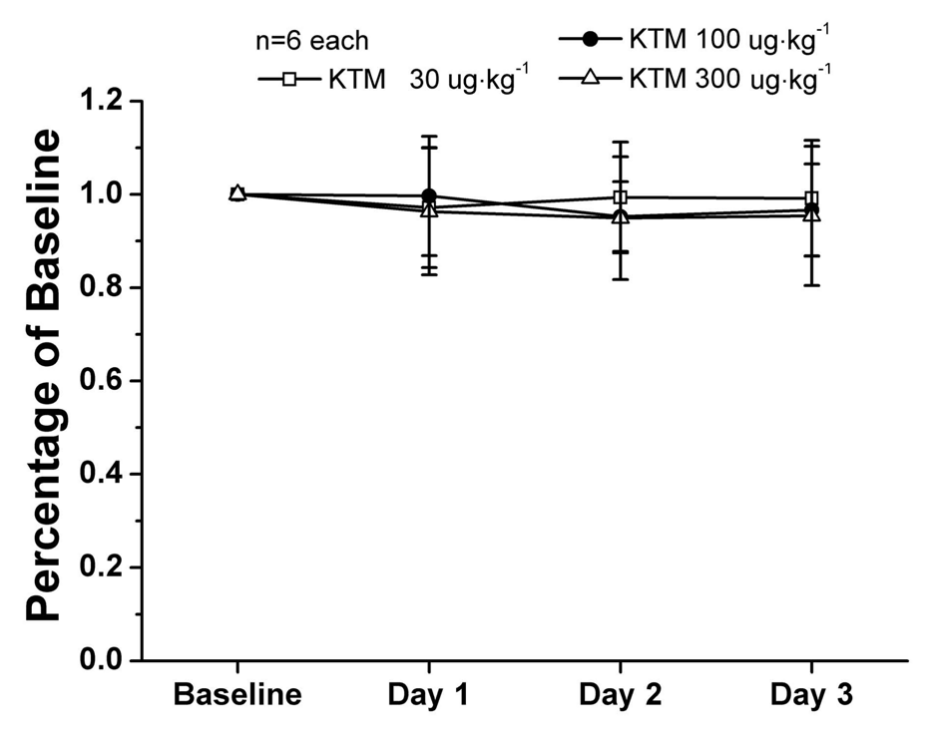
**Effects of ketamine on motor performance of rats in the rotarod test**. After recording baseline response, ketamine (30, 100 or 300 μg kg^-1^) was administered intrathecally and rotarod test was performed 30 min later once a day for 3 days, respectively. Compared with the baseline response, no statistical differences were obtained from rotarod test after intrathecal ketamine. There were six rats in each group. KTM: ketamine.

Nociceptive behavioral results could be easily influenced as a result of motor dysfunction. In order to assess whether the dosages of ketamine used in the present study (30, 100 and 300 μg kg^-1^) could produce impairment of motor functions, 18 otherwise experiment-free rats were assessed using a rotarod test. Intrathecal ketamine (30, 100 and 300 μg kg^-1^) did not affect the motor performance of rats 30 min after injection compared with their own baseline (Figure 3). Furthermore, repeated administrations did not influence motor function either.

### Ketamine attenuates SNL-induced mechanical allodynia in a dose-dependent manner

**Figure 4:**
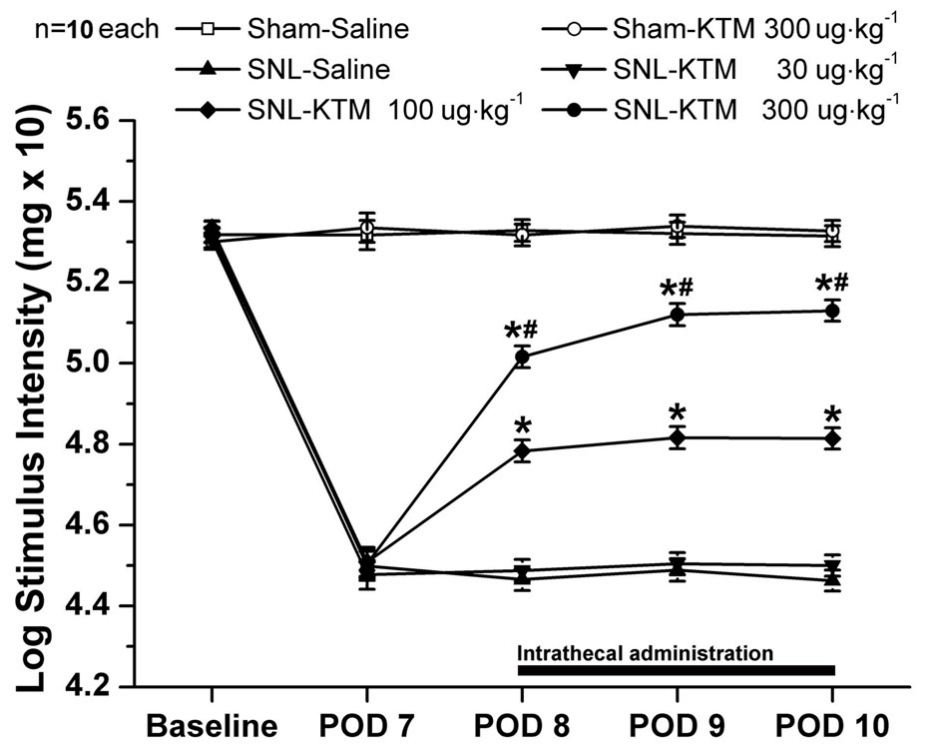
**Effects of intrathecal ketamine on SNL-induced mechanical allodynia**. SNL induced significant mechanical allodynia as shown by von-Frey tests. Intrathecal 300 μg kg^-1 ^ketamine did not change the normal pain threshold of the sham-operated group. Intrathecal ketamine (100 and 300 μg kg^-1^) showed an effective and reliable anti-allodynia effect in a dose-dependent manner on SNL-induced neuropathic pain, whereas, intrathecally 30 μg kg^-1 ^of ketamine did not influence pain threshold after SNL at all. Drugs were given intrathecally once a day from POD 8 to POD 10. *P < 0.05, compared with that of SNL-Saline group. ^#^*P *< 0.05, compared with that of SNL-ketamine 100 μg kg^-1 ^group. There were ten rats in each group. KTM: ketamine.

In order to verify the effects of intrathecal ketamine on SNL-induced neuropathic pain, ketamine was injected once a day with three different dosages and changes in PWT were observed 30 min after injection, from POD 8 to POD 10 (Figure 4).

SNL induced significant mechanical allodynia as shown in the SNL-Saline group. Compared with that of the SNL-Saline group, intrathecal injection of 30 μg kg^-1 ^ketamine did not influence PWT (Figure 4). Intrathecal ketamine (100 μg kg^-1^) elevated PWT significantly after administration (*P *< 0.05, compared with that of SNL-Saline). Furthermore, a higher dose of ketamine (300 μg kg^-1^) apparently elevated PWT (*P *< 0.05, compared with that of SNL-ketamine 100 μg kg^-1^) (Figure 4). However, neither high (300 μg kg^-1^) nor lower doses (30 and 100 μg kg^-1^, data not shown) of ketamine changed the basal threshold in the sham-operated group (Figure 4). These results demonstrate that intrathecal ketamine (30, 100 and 300 μg kg^-1^) produces an effective and reliable anti-allodynia effect in a dose-dependent manner on SNL-induced neuropathic pain.

### Effects of intrathecal ketamine on SNL-induced pJNK expression

**Figure 5:**
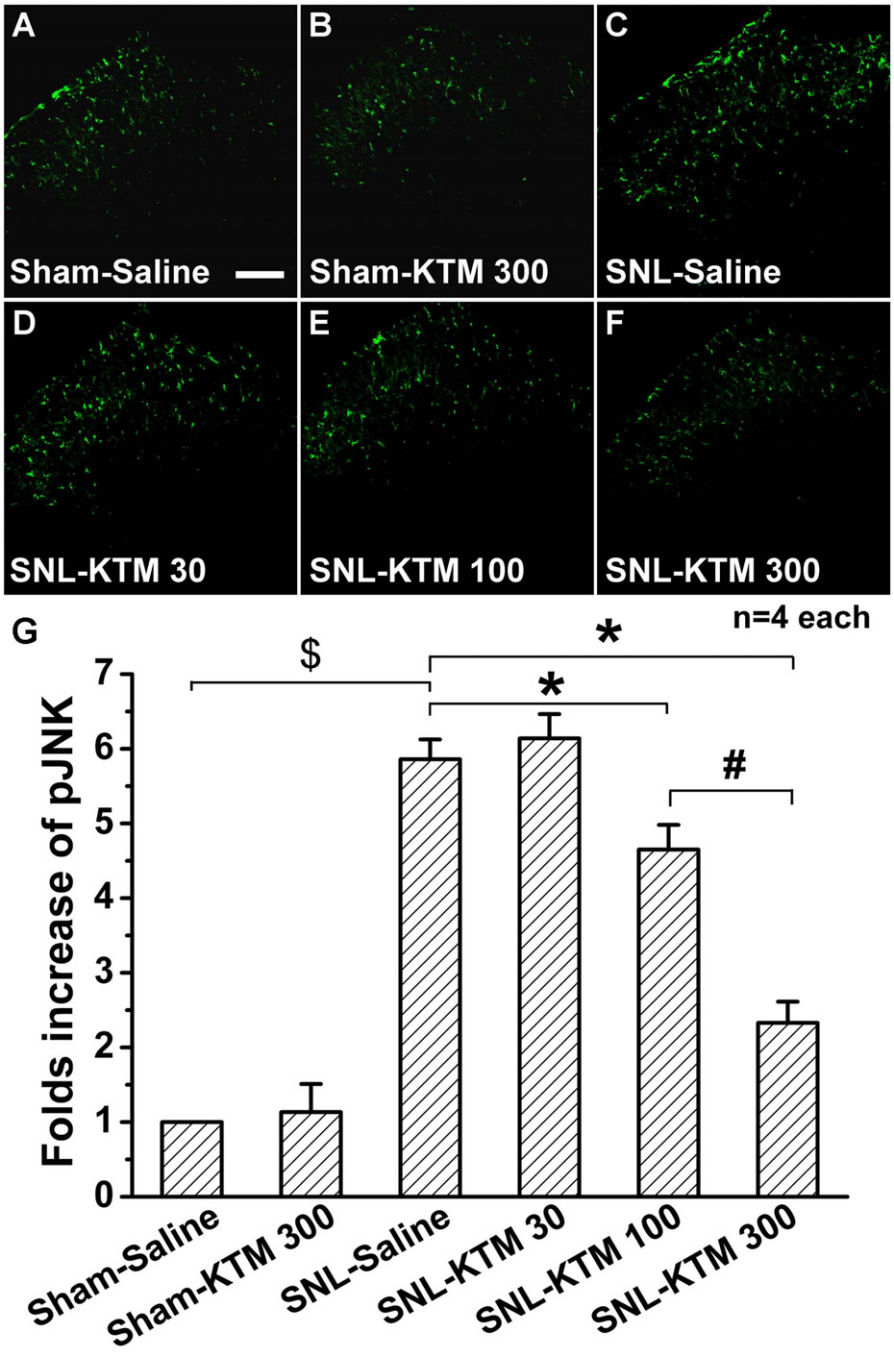
**Effect of intrathecal ketamine on SNL-induced pJNK expression**. Only a little pJNK expression could be detected in the Sham-Saline group (A), and intrathecal ketamine (300 μg kg^-1^) had no effect on pJNK expression in sham-operated rats (B). SNL induced significant pJNK expression (C), which could be suppressed by intrathecal ketamine in a dose-dependent manner (D-F). G, statistical analysis of the pJNK expressions after different treatments. *, # or $ each indicates statistically significant difference with *P *< 0.05 between groups. Four rats in each group. Scale bar: 100 μm. KTM: ketamine.

**Figure 6:**
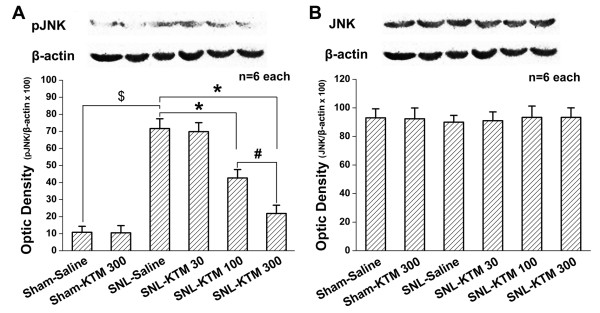
**Western blot quantification of JNK and pJNK expressions after different treatments**. A: Compared with that of Sham-Saline group, no difference could be detected in pJNK expression after intrathecal ketamine (300 μg kg^-1^) to sham-operated rats, whereas SNL apparently induced pJNK up-regulation. However, intrathecal ketamine down-regulated SNL-induced pJNK expression in a dose-dependent manner, which was in accord with the immunofluorescent histochemical staining result. B: Spinal JNK protein expression did not show any obvious difference after different treatments. *, # or $ each indicates statistically significant difference with *P *< 0.05 between groups. There were six rats in each group. KTM: ketamine.

Next, the effect of intrathecal ketamine on JNK phosphorylation was tested. Intrathecal ketamine (300 μg kg^-1^) did not show any effect on pJNK expression in sham operated rats compared with that of the Sham-Saline group (Figure 5:A, B), which was also verified by western blot (Figure 6A). SNL enhanced expression of pJNK in spinal dorsal horn (Figure 5C), which was increased about 5.62-fold in the SNL-Saline group compared with that of the Sham-Saline group (Figure 6A, *p *< 0.05). Intrathecal ketamine produced a dose-dependent effect on suppression of SNL-induced pJNK up-regulation in spinal dorsal horn (Figure 5:D-F, Figure 6A). Intrathecal 30 μg kg^-1 ^ketamine did not show any obvious effect on pJNK expression compared with that of the SNL-Saline group (Figure 5D, Figure 6A). However, the expression of pJNK was significantly attenuated to 59.54% of the SNL-Saline group by intrathecal 100 μg kg^-1 ^of ketamine (Figure 5E, Figure 6A, *P *< 0.05). Moreover, intrathecal 300 μg kg^-1 ^ketamine significantly down-regulated pJNK expression (Figure 5F), to just 30.46% that of the SNL-Saline group (Figure 6A, *P *< 0.05). Furthermore, intrathecal 300 μg kg^-1 ^ketamine showed stronger effects on pJNK expression compared with that of the SNL-ketamine 100 μg kg^-1 ^ketamine group (Figure 6A, *P *< 0.05). These results suggest that intrathecal ketamine can effectively inhibit pJNK up-regulation in a dose-dependent manner. However, neither SNL operation nor intrathecal ketamine (30, 100 and 300 μg kg^-1^) had any obvious effect on spinal JNK expression (Figure 6B). This result suggests that intrathecal ketamine only suppresses activation of the JNK pathway, without changing spinal expression of total JNK.

## Discussion

We have previously shown that intrathecal ketamine attenuates SNL-induced neuropathic pain and inhibits astrocytic activation in lumbar spinal dorsal horn [21]. However, the molecular mechanisms of how ketamine influences astrocytic function are unclear. In the present study we show that intrathecal ketamine significantly suppresses nerve injury-induced mechanical allodynia. More interestingly, the activation of JNK, which is selectively expressed in spinal astrocytes, could be inhibited by ketamine. Our results suggest that inhibiting activation of astrocytic JNK may play an important role in the analgesic effect of ketamine.

JNK is a member of the mitogen-activated protein kinase (MAPK) family, which has been shown to have a critical role in intracellular signal transduction. It has been confirmed that both JNK1 and JNK2 are constitutively expressed in the spinal cord; however, only phosphorylated JNK1 (pJNK1, active form) is increased after SNL [8]. Thus, for the present study, we focused on the phosphorylated form of JNK (pJNK1) after SNL and drug administration. Present results showed that JNK phosphorylation was highly up-regulated and specifically localized in spinal astrocytes. These results are in accord with a previous report that SNL induces a slow (>3 d) and persistent (>21 d) activation of JNK in spinal astrocytes [8].

Previous reports have confirmed that the JNK pathway is preferentially activated in spinal astrocytes [8,11,29,30], which is different from the pERK and p-p38 pathways at the spinal level after nerve injury [10,15]. It also has been validated that intrathecal administration of a JNK inhibitor can suppress spinal astrocytic activation and relieve neuropathic pain after SNL [8,31]. Present study showed that ketamine suppressed pJNK expression in a dose-dependent manner. Therefore, this may be the underlying molecular mechanism for ketamine to inhibit astrocytic activation and to relief mechanical allodynia, especially after nerve injury.

Ketamine has been reported to exert anti-inflammatory effects on macrophages stimulated with lipopolysaccharide (LPS) *in vitro *and *in vivo*, with involvement of toll-like receptors (TLRs) and MAPK pathways (JNK and ERK) [23,32]. Glial cells, the "immunocytes" of the CNS, somewhat like macrophages, have been confirmed to be involved in the immune response in neuropathic pain condition [33]. Furthermore, it has been reported that ketamine reduces LPS-induced tumor necrosis factor (TNF)-α, and prostaglandin E2 (PGE2) production in astrocyte cultures to inhibit some LPS-induced astrocytic inflammatory responses [22]. Therefore, intrathecal ketamine could attenuate SNL-induced mechanical allodynia by suppression of astrocytic JNK activation, which has been verified by the present research. However, how does ketamine function on astrocytic JNK activation after intrathecal injection?

Ketamine has been recognized as a nonselective NMDA receptors antagonist. A previous study has shown that astrocytic activation is markedly suppressed by treatment with the NR2B- (a subunit of NMDA receptor) selective antagonist ifenprodil in neuron-glia co-cultures, but not in purified astrocytes [34]. For this reason, blocking NMDA receptor on neurons might be an indirect method to suppress astrocytic activation with pJNK expression after intrathecal ketamine.

Evidence indicates that proinflammatory cytokines such as TNF-α, interleukin (IL)-1β, and IL-6 are primary activators of JNK pathway [12,35,36]. Activation of JNK pathways also can lead to production and release of multiple proinflammatory mediators [17,37], which form an excitatory feedback loop among glial cells and neurons resulting in enhanced excitability and plasticity [7,33,38,39]. Ketamine can exert anti-inflammatory effects in vitro and in vivo [40-43]. It is reported that ketamine can inhibit the effects of TNF-α on cellular signal transmission [43]. Moreover, it is also confirmed that ketamine can reduce astrocytic TNF-α expression and release [22], which may break down the positive feedback loop and alleviate the neuropathic pain. Consequently, suppression of proinflammatory factors expression after peripheral nerve injury could be an option for down-regulating pJNK expression by intrathecal ketamine.

In addition, accumulating studies have indicated that TLRs, expressed widely on spinal glia, are involved in the development of neuropathic pain after nerve injury [44-46]. It has been suggested that glial TLRs could be new targets for treatment of neuropathic pain [45]. TLRs in spinal cord may have a role in the early establishment of neuropathic pain after peripheral nerve injury [44,46]. A previous report indicated that TLR3 could mediate signals that induce proinflammatory cytokine and chemokine gene expression in astrocytes, which could be prevented by SP600125, a pharmacological inhibitor of JNK [37]. This report indicates that activation of JNK is required for TLR3-mediated proinflammatory cytokine and chemokine gene expression in astrocytes. Additionally, ketamine can inhibit proinflammatory gene expression, such as TNF-α, IL-1β, IL-6 and so on, possibly by suppressing TLRs-mediated signal-transduction [22,32,40,47]. Accordingly, ketamine might act on TLRs located on astrocytes and thus inhibit astrocytic JNK activation and, consequently, neuropathic pain.

## Conclusions

Given the important role of the astrocytic JNK pathway in neuropathic pain, inhibiting JNK activation could be useful in the treatment of neuropathic pain. The present study shows that intrathecal ketamine can down-regulate astrocytic pJNK expression and attenuate mechanical allodynia in a dose-dependent manner. Although more detailed mechanisms need to be further investigated and classified, the present work provides a potential strategy for treating neuropathic pain.

## Competing interests

The authors declare that they have no competing interests.

## Authors' contributions

XPM and HZ performed the animal surgery, carried out the Western blot study and drafted the manuscript. MZZ and YYW carried out the immunofluorescence and western blot. WW (Wei Wang) performed the behavioral test. WW (Wen Wang) participated in producing graphics and performed the statistical analysis. LXX and YQL conceived the study, and participated in its design and coordination. All authors read and approved the final manuscript.
